# ΔNp63α promotes Bortezomib resistance via the CYGB–ROS axis in head and neck squamous cell carcinoma

**DOI:** 10.1038/s41419-022-04790-0

**Published:** 2022-04-09

**Authors:** Peng Zhou, Caiyun Zhang, Xianmin Song, Dadong Zhang, Minhui Zhu, Hongliang Zheng

**Affiliations:** 1 Department of Otorhinolaryngology-Head and Neck Surgery, Changhai Hospital, Second Military Medical University, Shanghai, 200433 China; 2 Department of Otorhinolaryngology-Head and Neck Surgery, Affiliated Hospital of Xuzhou Medical University, Xuzhou, Jiangsu 221003 China; 3 3D Medicines Inc., Shanghai, 201114 China

**Keywords:** Predictive markers, Head and neck cancer

## Abstract

Bortezomib, a proteasome inhibitor, proved potent in the treatment of recurrent multiple myeloma or mantle cell lymphoma. However, slow progress was made when it was applied to treat solid tumors. We discovered that different head and neck squamous cell carcinoma (HNSCC) cell lines had significantly different sensitivities to bortezomib, and also demonstrated that individual relatively sensitive HNSCC cell lines had fewer ΔNp63α expressions. Based on these findings, we speculated that ΔNp63α may be a key factor in the resistance of HNSCC cells to bortezomib. ΔNp63α knockdown made HNSCC more sensitive to bortezomib, while ΔNp63α overexpression made it more resistant. RNA sequencing (RNA-seq) analysis of ΔNp63α-knockdown cells revealed clear alterations in the subset of genes that were associated with oxidative stress and antioxidant defense. The gene CYGB was downregulated significantly. CHIP-seq detection showed that CYGB was the transcriptional regulatory site of ΔNp63α. CHIP-PCR showed evidence of ΔNp63α binding. The detection of the dual-luciferase reporter gene demonstrated that ΔNp63α significantly enhanced the CYGB promoter activity. Furthermore, we confirmed that CYGB plays a role in clearing excess ROS induced by bortezomib to inhibit HNSCC apoptosis. Consequently, ΔNp63α regulated the expression of CYGB in HNSCC. CYGB was the target of transcription regulation of ΔNp63α. It reduced apoptosis by clearing excess ROS produced by bortezomib, and thus exerted drug resistance.

## Introduction

According to GLOBECAN, in 2018 alone, there were 705,781 new cases of head and neck cancer (HNC) worldwide, and 358,144 patients died of HNC, accounting for 3.9% incidence of and 3.7% mortality from systemic malignancies [1]. HNSCC is the most common type of HNC. At present, about 60% of patients with HNSCC have undergone regional metastasis or are in the advanced clinical stage at the time of consultation[2]. For these patients, traditional treatment has not increased the long-term survival rate. Proteasome inhibitors are increasingly becoming an important adjuvant therapy. They can specifically recognize and kill tumor cells and have potential application prospects. In the past ten years, the application of proteasome inhibitors in the treatment of hematological malignancies has greatly expanded [3–5]. However, slow progress has been achieved in the treatment of solid tumors. There may be numerous reasons for this, but the inherent resistance mechanism in solid tumor cells is probably the most important.

In general, mutations, overexpression, or persistent activation of certain proteins in a cell may contribute to drug resistance. P63 is located on chromosome 3; it belongs to the same family as p53 and can regulate p53 downstream gene expression [6]. The ΔNp63α subtype is the most common isoform type, which is highly expressed specifically in most HNSCC [7]. Previous studies have demonstrated that highly expressed ΔNp63α can promote cell survival and is a significant prognostic marker for HNSCC [8]. However, the of role ΔNp63α in bortezomib resistance was seldom known.

Cytoglobin (CYGB) is a member of the globin family. Other globin includes hemoglobin, myoglobin, and cerebrin, and their distributions have obvious tissue specificity—they are distributed in red blood cells, muscles, and nerve tissues respectively—but CYGB can be expressed in multiple tissues and organs. Like other globin, CYGB has a compact helical conformation and can reversibly bind diatomic gas molecules such as O_2_, NO, CO [9]. Although research on CYGB is still ongoing, and some of its biological functions are still being explored, it has been shown to have antioxidant functions. CYGB expression can increase in the presence of hypoxia, oxidative stress, or fibrosis stimuli. Studies have shown an increase in intracellular CYGB expression when it is exposed to oxidants. For example, in neuroblastoma cells, CYGB levels increased after stimulation by calcium ions, alginic acid, heat shock, or high osmotic pressure [10].

In addition, after treatment with oxidants, the ability of highly expressed CYGB in primary hematopoietic stem cells or fibroblasts to scavenge reactive oxygen species (ROS) improved, so DNA damage was significantly reduced and the survival rate increased [11]. Other studies have confirmed that CYGB in squamous epithelial cells has a positive effect of clearing excess ROS protective cells [12, 13]. Here, we report that CYGB removed excessive intracellular ROS within limits in HNSCC after treatment with bortezomib. The CHIP-seq results indicated that the CYGB promoter was under ΔNp63α transcriptional control, and that ΔNp63α, through its target CYGB, promoted HNSCC cell survival under induced oxidative stress and showed drug resistance.

## Results

### Verify the expression level of ΔNp63α in HNSCC relationship with bortezomib sensitivity

**Fig. 1:**
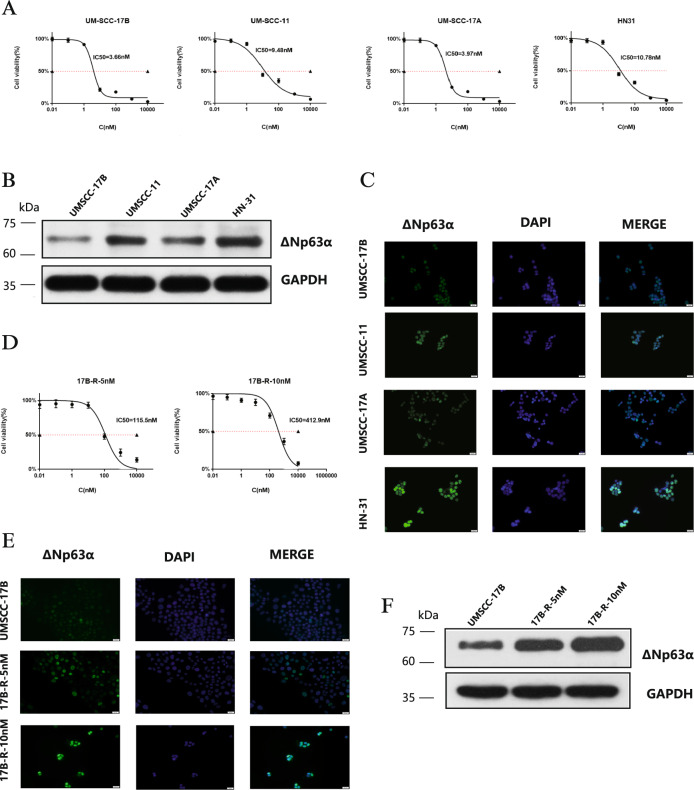
The bortezomib sensitivity of HNSCC is associated with the expression level of ΔNp63α. **A** UMSCC-17B, UMSCC-11, UMSCC-17A, and HN31 cells were treated with increasing concentrations of bortezomib (0.01–10000 nM) for 24 h, viable cells were quantified using the CCK-8 assay. Each data point represents the means ± SD of three separate experiments. **B**. Western blot was performed to analyze ΔNp63α proteins levels of UMSCC-17B, UMSCC-11, UMSCC-17A, and HN31 cells. **C**. Four cells were observed for ΔNp63α by immunostaining with antibody (green). Nuclei are counterstained with DAPI (blue). Scale bar: 20 μm. **D**. After “step-wise” inducing drug-resistance cell culture, 17B-R-10nM drug-resistant cell line that can proliferate in culture medium containing 10 nM bortezomib was obtained. IC50 of 17B-R-5nM and 17B-R-10nM were calculated using, CCK-8 assay. **E**. UMSCC-17B, 17B-R-5nM, and 17B-R-10nM were observed for ΔNp63α by immunostaining with antibody (green) Scale bar: 20 μm. **F**. Altered expressions of ΔNp63α were evaluated by western blot analysis in UMSCC-17B, 17B-R-5nM, and 17B-R-10nM.

To compare bortezomib sensitivity among the four HNSCC cell lines, we tested IC50 values of them. As shown in Fig. 1A, HN31 and UMSCC-11 strains were more resistant to bortezomib than UMSCC-17A and UMSCC-17B strains. We also investigated the expression of protein ΔNp63α of the four strains. According to the western blot test (Fig. 1B and Supplementary Fig. WB-1) and immunofluorescence (Fig. 1C) results, expressions of ΔNp63α in HN31 and UMSCC-11 cells were significantly higher than the expressions in UMSCC-17A and UMSCC-17B cells. The above four strains demonstrated a clear correspondence between ΔNp63α expression and bortezomib drug sensitivity: cell lines with high ΔNp63α expression (HN31 and UM SCC-11) were relatively resistant to bortezomib, while cell lines with low Δ Np63α expression (UMSCC-17A and UMSCC-17B) were relatively sensitive to bortezomib.

After “step-wise” inducing drug resistance, we obtained two drug-resistant cell lines: 17B-R-5nM and 17B-R-10nM. IC50 values of 17B-R-5nM cells and 17B-R-10nM cells were 412.9 nM and 115.5 nM respectively (Fig. 1D). Immunofluorescence (Fig. 1E) and western blot showed that (Fig. 1F and Supplementary Fig. WB-1), the expression of ΔNp63α increased substantially with the drug resistance index.

Taken together, our results indicated that increased ΔNp63α expression is involved in bortezomib resistance in HNSCC.

### ΔNp63α modulates bortezomib response in vitro

**Fig. 2:**
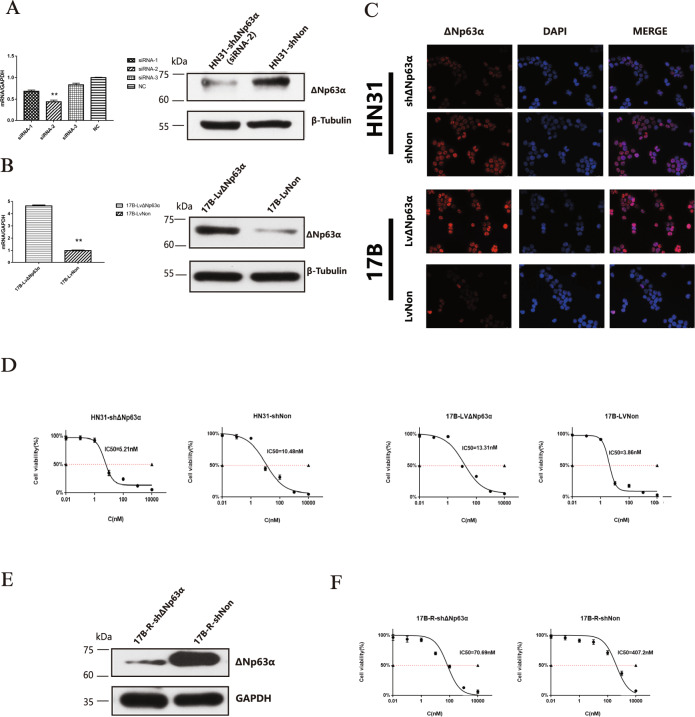
ΔNp63α knockdown causes HNSCC more sensitive to bortezomib and vice versa for ΔNp63α overexpression. **A**. After three interfering sequence lentiviruses were transfected into HN31 cells, ΔNp63α mRNA levels of these three cell strains were detected by qPCR, indicating that the knockdown efficiency of sequence 2 was the most obvious. And the results were verified by western blot. **P < 0.01. **B**. UMSCC-17B cell was transfected with lentivirus and overexpressed ΔNp63α, and it was verified by qPCR and Western blot that the stably transfected strain had an obvious overexpression effect. **C**. HN31-shΔNp63α and 17B-LvΔNp63α were observed for ΔNp63α by immunostaining with antibody (red). Nuclei are counterstained with DAPI (blue). Scale bar: 20 μm. **D**. HN31-shΔNp63α, HN31-shNon, 17B-LvΔNp63α, and 17B-LvNon cells were treated with increasing concentrations of bortezomib (0.01–10000 nM) for 24 h, and IC50 was calculated using CCK-8 assay. **E**. 17B-R (17B-R-10nM) cells were also transfected with shRNA to knockdown ΔNp63α and the result was verified by western blot. **F** IC50 of 17B-R- shΔNp63α and 17B-R-shNon was calculated using the CCK-8 assay.

To investigate the effect of ΔNp63α protein on bortezomib sensitivity, HN31 cells were transfected with shΔNp63α plasmids for ΔNp63α knockdown, while UM-SCC-17B cells were transfected with LvΔNp63α plasmids for ΔNp63α overexpression. PCR and Western blot detection were used to verify the efficiency of ΔNp63α knockdown or overexpression (Fig. 2A and B, Supplementary Fig. WB-1). Immunofluorescence (Fig. 2C) showed a dramatic decrease of the ΔNp63α protein level in HN31-shΔNp63α compared to HN31-shNon and a significant increase of ΔNp63α protein level in 17B-LvΔNp63α compared to the control group. The results of CCK-8 test (Fig. 2D) showed that HN31 cells became more sensitive to bortezomib after ΔNp63α knockdown, while 17B cells turned into more resistant to bortezomib after ΔNp63α overexpression. Furthermore, 17B-R (17B-R-10nM) cells were transfected with shΔNp63α plasmids and the efficiency was checked by Western blot detection (Fig. 2E and Supplementary Fig. WB-1). After ΔNp63α knockdown, 17B-R cells became more sensitive to bortezomib (Fig. 2F). These results confirmed that ΔNp63α protein rendered HNSCC cells more resistant.

### ΔNp63α exerts a promotional effect on tumorigenesis and drug-resistance in vivo

**Fig. 3:**
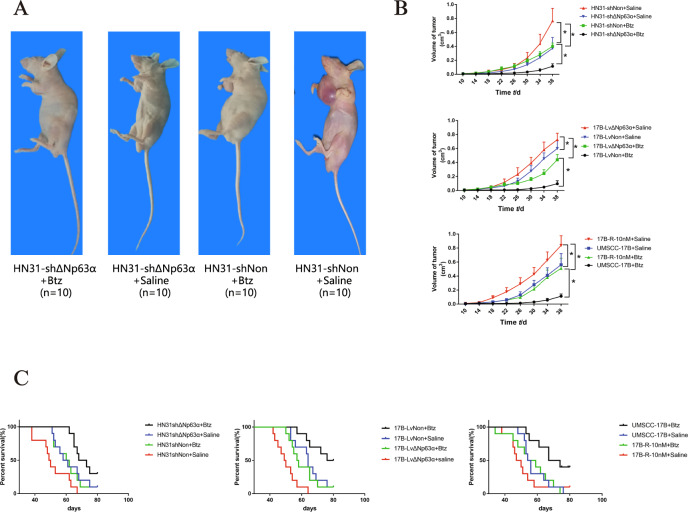
ΔNp63α exerts a promotional effect on tumorigenesis and drug-resistance in vivo. **A**. BALB/c nude mice subcutaneous HNSCC tumors were given tail vein injections of bortezomib (Btz) or saline. The injection dose of bortezomib injection is 0.3 mg/kg, and they were injected every three days for 6 consecutive weeks. Mice of groups (HN31-shΔNp63α + Btz, HN31-shΔNp63α + Saline, HN31-shNon + Btz, and HN31-shNon + Saline) of the 38th day were shown above. **B**. Larger diameters and smaller diameters of the tumors were measured and the volume of the tumors was calculated in the following groups (HN31-shΔNp63α + Btz, HN31-shΔNp63α + Saline, HN31-shNon + Btz, HN31-shNon + Saline, 17B-LvΔNp63α + Btz, 17B-LvΔNp63α + Saline, 17B-LvNon + Btz, 17B-LvNon + saline, 17B-R-10nM + Btz, 17B-R-10nM + Saline, 17B-P + Saline, and 17B-P + Btz). * *P* < 0.05. **C**. Comparisons of survival curves were performed in these groups.

We examined the tumorigenesis of HNSCC cells in vivo to further determine the effect of ΔNp63α on bortezomib resistance. HN31-shΔNp63α cells, HN31-shNon cells, 17B-LvΔNp63α cells, 17B-LvNon cells, UMSCC-17B cells, and 17B-R-10nM cells were injected subcutaneously in nude mice (*n* = 10) combined with bortezomib or saline treatment as a control to investigate the effect of ΔNp63α on bortezomib therapy. Following the treatment of bortezomib, silencing ΔNp63α decreased the growth of HN-31 cells in vivo (Fig. 3A and B), whereas forced ΔNp63α expression enhanced significantly the growth of UMSCC-17B (Fig. 3B). Additionally, the growth of tumors in the UMSCC-17B group was much slower than17B-R-10nM that in the group with bortezomib treatment (Fig. 3B). As shown in Fig. 3C, silencing ΔNp63α significantly enhanced the survival of mice with bortezomib (*P* = 0.035). In contrast, introducing ΔNp63α into 17B cells significantly decreased the survival of mice following bortezomib treatment (*P* = 0.011). Furthermore, under the treatment of bortezomib, the survival of UMSCC-17B group was significantly higher than 17B-R-10 nM group (*P* = 0.012). These results clearly suggested that ΔNp63α expression is important for treating HNSCC with bortezomib and ΔNp63α exerts a promotional effect on drug-resistance in vivo.

### ΔNp63α knockdown changed the landscape of oxidative stress

**Fig. 4:**
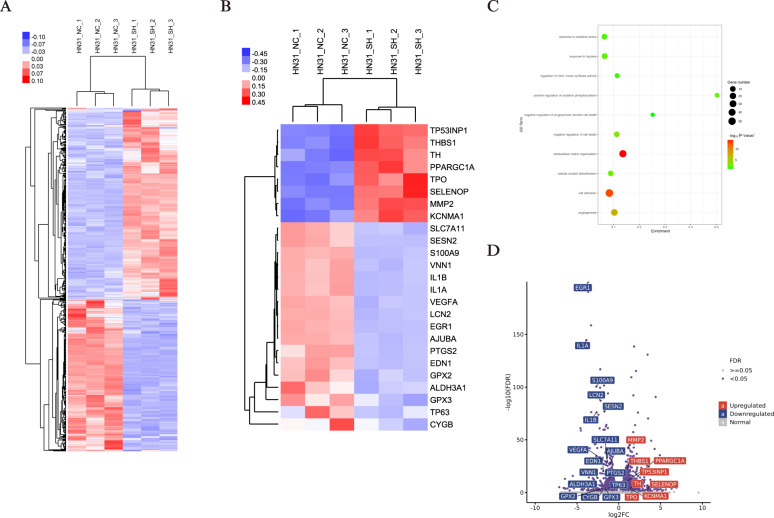
RNA-seq shows ΔNp63α knockdown changed the landscape of oxidative stress. **A**. Heatmap of all differentially expressed genes between HN31-shΔNp63α cells and HN31-shΔNon cells (log_2_ fold change >1 or < -1, FDR < 0.05). **B**. Heatmap of differentially expressed genes associated with oxidative stress and antioxidant defense. **C**. Gene Ontology (GO) was analyzed and demonstrated for identifying and visualizing enriched GO terms. **D**. Scatter plot of differentially genes after ΔNp63α knockdown. Blue and red indicate the downregulated and variation upregulated, respectively.

In order to investigate the differential gene expression after ΔNp63α knockdown, we successfully conducted transcriptomic RNA-seq for HN31-shΔNp63α cells and HN31-shΔNon cells. In total, we identified 449 up-regulated and 358 down-regulated genes (Fig. 4A). GO analyses showed significant subsets of genes in GO terms such as oxidative stress, hypoxia, regulation of nitric-oxide synthase activity, and cellular oxidant detoxification (Fig. 4C). As shown in Fig. 4B and D, the upregulated genes included TP53INP1, THBS1, TH, PPARGC1A, TPO, MMP2, while the downregulated genes included GPX2, GPX3, PTGS2, CYGB, VEGFA, EGR1, EDN1, LCN2. Among these antioxidants (GPX2, GPX3, PTGS2, CYGB), GPX2 has already been described as a unique target gene of p63 [14]. Here, we focus on CYGB, which exerts a promotional effect on clearing excess ROS to protect squamous epithelial cells [12].

### CYGB promoter methylation status in HNSCC

Since CYGB was found as highly methylated in upper aero-digestive tract squamous cancer [15], and aberrant methylation of CpG islands may cause silencing of certain gene [16], it’s necessary to test CYGB promoter methylation status in HN31 cells and UMSCC-17B cells. Bisulfite sequencing PCR analysis was carried out and an average methylation index (MtI) was calculated from the mean of the CpG sites evaluated. Two CpG-rich CYGB promoter regions were presented in Supplementary Fig. S4A. As shown in Supplementary Fig. S4B and C, methylation rate of HN-31 in the first CpG island was 0% and that in the second CpG island was 0.6%. The MtI of HN-31 was 0.3%. Methylation rate of UMSCC-17B in the first CpG island was 1% and that in the second CpG island was 0.6%. The MtI of UMSCC-17B was 0.8%. According to the previous study, a minimum threshold MtI of approximately 25% was considered as hypomethylation of the CpG island [17]. So, our results confirmed that hypomethylation of the CpG island within the CYGB promoter region was observed in these two HNSCC lines.

### Effect of ΔNp63α on ROS induced by bortezomib

**Fig. 5:**
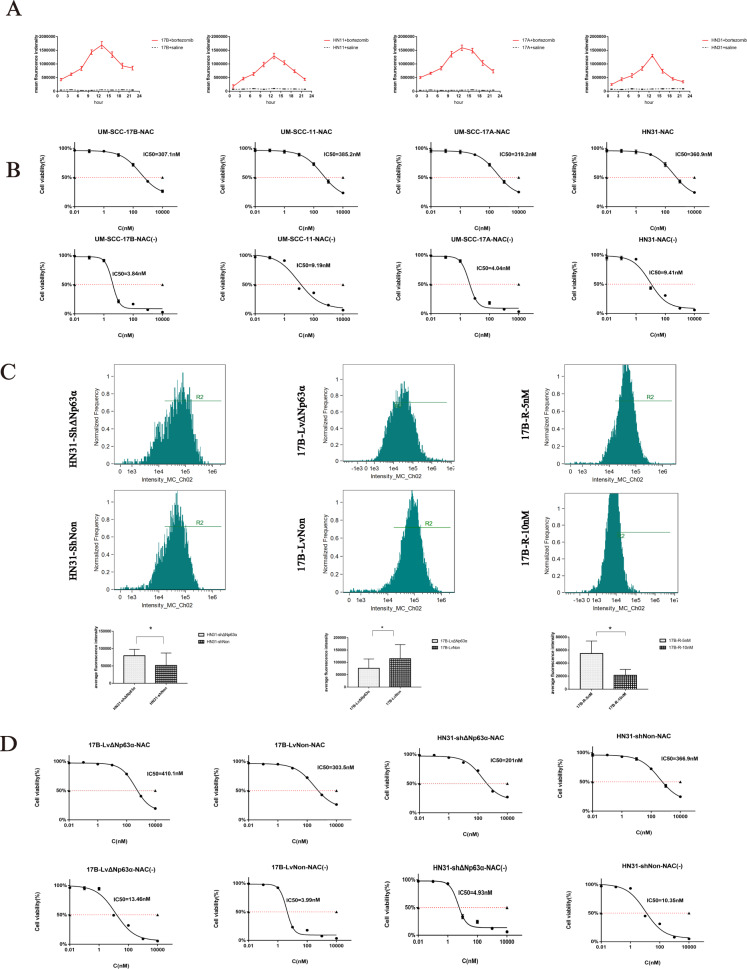
ΔNp63α regulates HNSCC cell to bortezomib resistance via ROS. **A**. Four strain cells were exposed to 10 nM bortezomib or to the same volume of PBS solution as a control. At the indicated times, cells were taken from culture and incubated in the presence of 10uM DCFH-DA for the determination of ROS generation. **B**. NAC was used to pre-protect UMSCC-17B, UMSCC-11, UMSCC-17A, and HN31 before exposure to bortezomib. IC50 was calculated using the CCK-8 assay. **C**. The histograms showed the DCF fluorescence intensity for evaluating ROS levels following bortezomib treatment (4 h) in HNSCC cells. The values reported are the mean±sd of three independent transfections. Comparisons were carried out between HN31-shΔNp63α and HN31-shNon, 17B-LvΔNp63α and 17B-LvNon, and 17B-R-5nM and 17B-R-10nM. *P < 0.01. **D**. NAC was used to pre-protect 17B-LvΔNp63α and HN31-shΔNp63α before exposure to bortezomib. IC50 was calculated using the CCK-8 assay.

The study [18] has shown that ROS generation is necessary for the initiation of bortezomib-induced apoptosis. So, we proceeded to examine ROS levels of four HNSCC cells after treating them with bortezomib. UMSCC-17B, UMSCC-11, UMSCC-17A, and HN31 cells exposed to 10 nM bortezomib for the indicated time were examined for changes in ROS generation using DCFH-DA probes (Fig. 5A). The ROS levels began to increase gradually just after 1 hour, peaked at the 13th hour, and then decreased. Meanwhile, after treatment with NAC(N-Acetyl-L-cysteine), the bortezomib IC50 value of four HNSCC cell lines increased significantly (Fig. 5B). These results confirmed that HNSCC produced a large amount of ROS after exposure to bortezomib and NAC antioxidant treatment protected HNSCC cells against bortezomib.

To further explore the effect of ΔNp63α on ROS induced by bortezomib, we tested ROS levels of HN31shΔNp63α, 17B-LvΔNp63α, and 17-R cells (Fig. 5C). After been treated with bortezomib (4 h), silencing ΔNp63α significantly enhanced the expression of ROS in HN31shΔNp63α cells, whereas introducing ΔNp63α into 17B cells decrease the expression of ROS. In addition, the fluorescence intensity of 17B-R-5nM cells was much more than those of 17B-R-10nM cells. We also tested the IC50 values of these cells (Fig. 5D). And the results confirmed that NAC rendered the cells more resistant to bortezomib.

Generally, our data suggested that ΔNp63α played a role in decreasing levels of ROS induced by bortezomib.

### ΔNp63α can regulate the expression of CYGB

**Fig. 6:**
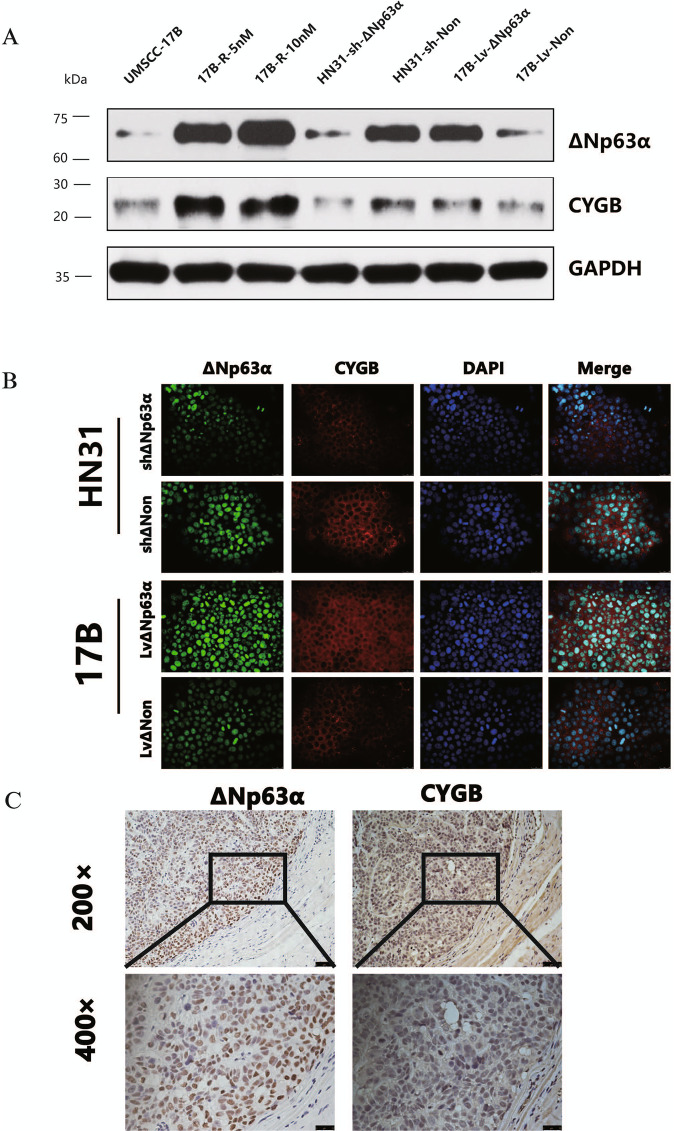
ΔNp63α can regulate the expression of CYGB. **A**. Western-blot was performed to analyze ΔNp63α and CYGB proteins levels of UMSCC-17B 17B-R-5nM, 17B-R-10nM, HN31-shΔNp63α, HN31-shNon, 17B-LvΔNp63α, and 17B-LvNon. **B**. Four strains (HN31-shΔNp63α, HN31-shNon, 17B-LvΔNp63α and 17B-LvNon) were observed for ΔNp63α (green) and CYGB (red) by immunostaining with antibodies. Nuclei were counterstained with DAPI (blue). Scale bar: 20 μm. **C**. Immunohistochemistry of nude mice tumors was performed to show the location and intensity of ΔNp63α and CYGB.

To investigate the correlation between protein CYGB and ΔNp63α expression in HNSCC, we performed Western blot and immunofluorescence staining on UMSCC-17B, 17B-R-5nM, 17B-R-10nM, HN31-shΔNp63α, HN31-shNon, 17B-LvΔNp63α, and 17B-LvΔNon cells. Western blot results (Fig. 6A and Supplementary Fig. WB-2) revealed that CYGB expression significantly decreased when ΔNp63α was knocked down, and CYGB was upregulated with overexpression of ΔNp63α. Furthermore, for the acquired resistance cells, the expressions of CYGB and ΔNp63α increased with the drug resistance index (Fig. 6A). Results of immunofluorescence were consistent with those of western blot (Fig. 6B and Supplementary Fig. S6).

We also investigated the expressions of the proteins CYGB and ΔNp63α in tumors isolated from nude mice by immunohistochemistry. ΔNp63α immunostaining was detected in the nucleus, while CYGB immunostaining was identified in the cytoplasm mostly (Fig. 6C). Taking into account the staining intensity and percentage of cells that stained at this intensity, scoring on CYGB and ΔNp63α expressions were calculated by the semi-quantitative H-score method. The results suggested a moderate positive correlation between the two proteins (Supplementary Table 1).

Taken together, our results indicated that ΔNp63α can regulate the expression of CYGB.

### ΔNp63α regulates ROS of HNSCC cells via CYGB to affect bortezomib resistance of HNSCC cells

**Fig. 7:**
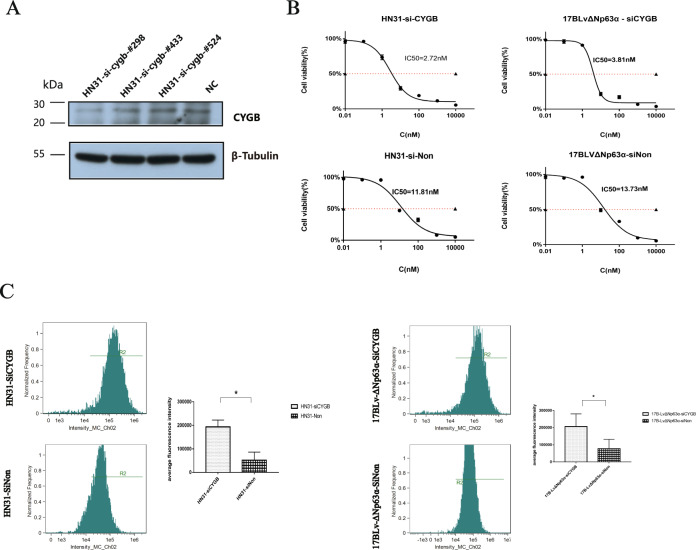
ΔNp63α regulates ROS of HNSCC cells via CYGB to affect drug resistance of HNSCC cells. **A**. The HN31 cell line was transfected with siRNA. After 24 h of transfection, Western-Blot verified the CYGB expression efficiency of three sequences (#298, #433, and#524). **B**. IC50 was calculated in HN31-siCYGB, HN31-siNon, 17BLvΔNp63α-siCYGB and 17BLvΔNp63α-siNon using CCK-8 assay. **C**. The histograms showed the DCF fluorescence intensity for evaluating ROS levels after bortezomib treatment (4 h) in HNSCC cells. Comparisons were performed between groups of HN31-siCYGB and HN31-siNon and between groups of 17BLvΔNp63α-siCYGB and 17BLvΔNp63α-siNon. *P < 0.01.

To investigate the effect of the protein CYGB on ROS induced by bortezomib, siRNA was transfected into HN-31 cells for CYGB knockdown. Western-blot test was performed to check the efficiency of CYGB knockdown and sequence #298 was selected (Fig. 7A and Supplementary Fig. WB-2). CYGB knockdown rendered HN31-siCYGB more sensitive to bortezomib (Fig. 7B). When exposed to bortezomib, HN31-siCYGB cells produced more amount of ROS than the control group (Fig. 7C). Moreover, when 17B-LvΔNp63α-siCYGB was exposed to bortezomib, the level of ROS increased (Fig. 7C). The IC50 value of 17B-LvΔNp63α-siCYGB decreased from 13.73 to 3.81 nM (Fig. 7B). Thus, knockdown of CYGB strengthened the effect on sensitivity to bortezomib.

### CYGB is a direct transcriptional target of ΔNp63α

**Fig. 8:**
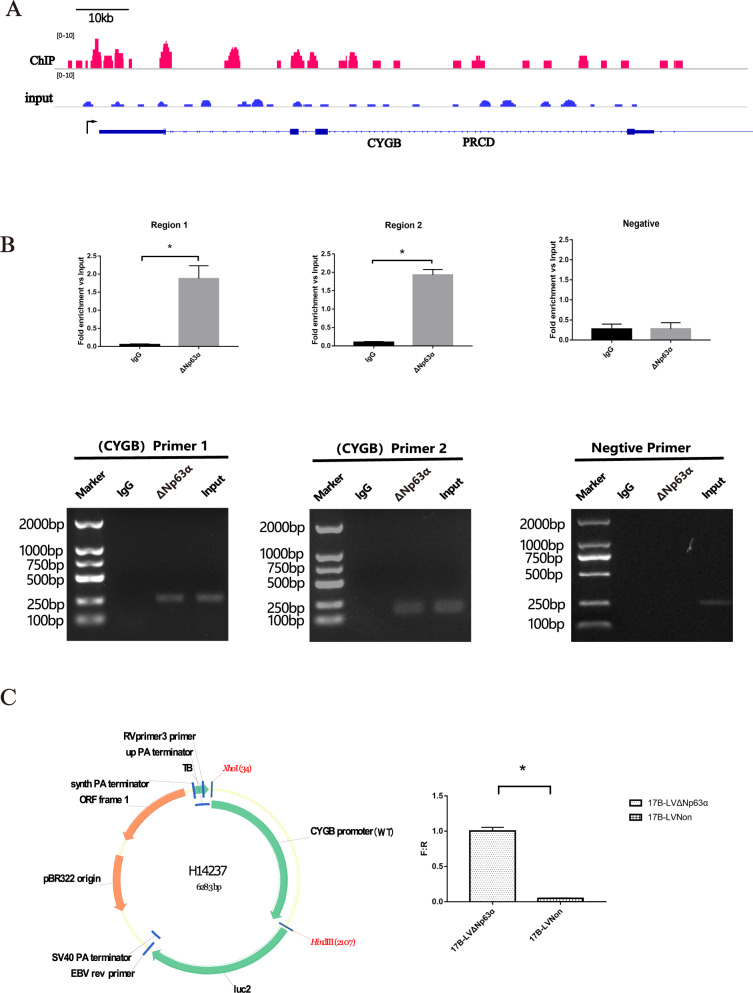
CYGB is a direct transcriptional target of ΔNp63α. **A**. ChIP analysis of the human regulatory region of CYGB was conducted by purifying chromatin from HN31 cells followed by immunoprecipitation with p63α antibody. ChIP-seq was conducted to demonstrate Peak in Chromosome 17. **B** ChIP analysis of the regulatory region of CYGB was conducted by purifying chromatin from HN31 cells followed by immunoprecipitation with ΔNp63 antibody. *P < 0.01 **C** Lipo3000 was used to simultaneously transfect CYGB plasmid and renilla plasmid into 17B-LvΔNp63α cells and 17B-LvNon cells. Firefly fluorescent substrate and renilla fluorescent substrate to detect the fluorescence intensity, Firefly fluorescent substrate and renilla fluorescent substrate were mixed to detect the fluorescence intensity. *P < 0.01.

To demonstrate that CYGB is a transcriptional target of ΔNp63α, CHIP-seq was performed on HN31 cells. After the sequencing data were obtained, we compared them with the reference genes using the Bowtie program, and the total number of matched reads bound to ΔNp63α was 3365491. The peak signal value over the recorded region was located in chromosome 17, and the ratio of Focus Ratio to Region Size was 0.914. There was a combination region of about 300 bp located 2.5–5 kb upstream from the transcription start site (Chr17:74536575-74536895) (Fig. 8A). Furthermore, we performed PCR with primer pairs designed to amplify sequences that included Region 1 and Region 2 elements (Fig. 8B). These regions showed evidence of ΔNp63α binding.

We also considered the effect of ΔNp63α on the CYGB promoter regulation using a luciferase assay (Fig. 8C). The promoter was cloned into the pGL4.10 H352 vector, and this construct was co-transfected in the 17B-LvΔNp63α and 17B-LvNon cells. We observed a significant (20-fold) increase in the luciferase activity in 17B-LvΔNp63α cells compared to 17B-LvNon cells. The data obtained showed that the regions contributed to promoter transcriptional activity.

We concluded that CYGB was the target of transcription regulation of ΔNp63α and ΔNp63α exerts a promotional effect on bortezomib resistance via CYGB-ROS axis.

## Discussion

Bortezomib has been confirmed to exert powerful cytotoxicity to NCI panel 60 cells lines of different cancers [19]. However, it was only approved for the treatment of multiple myeloma (2003) and a third-line treatment for a relapsed and refractory disease (2008) by FDA. Bortezomib seems to have nothing to do with solid tumors in clinical practice. We focus on bortezomib resistance HNSCC cell lines to explore the potential mechanisms.

We examined four HNSCC strains toxicity of bortezomib and protein ΔNp63α expressions and found that bortezomib sensitivity and ΔNp63α expressions were closely related. Generally speaking, overexpression of certain proteins in cells may promote the resistance of the cells to certain drugs. These proteins can activate or inhibit certain cell pathways so that tumor cells do not undergo apoptosis under the pressure of the drugs, thereby reducing the effectiveness of the drugs. Catherine BA et al. confirmed that the high expression of ΔNp63α promoted the survival of HNSCC in cisplatin [20]. In the current study, knockdown of ΔNp63α significantly rendered HNSCC more sensitive to bortezomib, while overexpression of ΔNp63α significantly made HNSCC more resistant to bortezomib. Our results in vitro and vivo suggested that ΔNp63α plays a crucial role in the resistance of HNSCC to bortezomib. That ΔNp63α promotes drug resistance has been demonstrated in cancer cell resistance to cisplatin [21, 22]. As we know, the ΔN variant of p63 has transcriptional activity [23, 24]. It also has been described that ΔNp63 regulated genes responsible for regulation of ROS and metabolism such as GPX2 [14], iRHOM2 [25], and REDD1 [26]. In the current study, we performed RNA-seq to analyze gene expression profiles of ΔNp63α knockdown cells and control cells. These results further implied that ΔNp63α contributed to key functions in oxidative stress and antioxidant. CYGB was significantly downregulated after ΔNp63α knockdown. A previous report has suggested that CYGB promoters are highly methylated in tumor cells with low expressions of CYGB [15]. However, our data suggested a significantly low rate of methylation in the two HNSCC cells. Based on these findings, we speculated that ΔNp63α exerted a novel molecular mechanism for bortezomib resistance.

It is acknowledged that endoplasmic reticulum stress induces ROS with the treatment of proteasome inhibition [18, 27–30]. Here, our results confirmed that bortezomib produced a large amount of ROS after acting on HNSCC cells. When NAC, an exogenous antioxidant, was utilized to pre-protect cells, the ROS produced decreased significantly under the same conditions. Additionally, HNSCC cells pretreated with NAC became resistant to bortezomib. These data revealed that ROS played a vital part in bortezomib-induced apoptosis and NAC promoted the survival of cells with bortezomib in HNSCC. These were in line with the results reported previously [18, 31]. Excessive ROS was cytotoxic and could activate the caspase signaling pathway [28], thereby increasing the sensitivity of bortezomib.

The effect of ΔNp63α expression on bortezomib-induced ROS levels showed that high expressions of ΔNp63α caused bortezomib to induce relatively fewer ROS, while low expressions of ΔNp63α did the opposite. In addition, it was verified that CYGB disposed of a protective effect on protection against oxidative stress in HNSCC. Our results showed that CYGB knockdown by siRNA significantly enhanced the ROS expression level and rendered the cells more sensitive to bortezomib. CYGB played a crucial role in ROS scavenging to protect HNSCC from bortezomib triggered by oxidative stress. Furthermore, the knockdown of CYGB expression in cell lines upregulated in ΔNp63α by lentivirus-transfect reduced the level of ROS, which suggested that ΔNp63α eliminated excess ROS by regulating CYGB. A study has shown that silencing the expression of CYGB in the SHSY5Y cell line exposed to oxidative stress could reduce the clearance of ROS, inhibit cell proliferation, and promote apoptosis [32]. This is in line with our results: CYGB showed significant antioxidant properties in cell experiments. Some studies speculate that the antioxidant effect of CYGB is similar to that of peroxidase and superoxide dismutase [33, 34]. However, the precise molecular mechanism underlying the anti-oxidative function is still poorly understood.

In addition to its anti-oxidation effect on ROS, CYGB has been found to be closely related to cancer, but whether CYGB is an oncogene or a tumor suppressor gene is controversial [35–38]. These studies suggested that CYGB may have a two-way regulatory role in the occurrence of tumors. Whether the expression of CYGB is upregulated or downregulated depends on the type of tumor cell, the tumor stage, and the tumor microenvironment.

In the current study, ΔNp63α knockdown decreased the expression of CYGB in HN-31cells, whereas forced expression of ΔNp63α enhanced the level of CYGB in UMSCC-17B. We found that CYGB is the transcription target of ΔNp63α using CHIP-seq, and verified it by PCR. Furthermore, the detection of the activity of the ΔNp63α-regulated CYGB promoter by the dual-luciferase reporter gene upheld the existence and function of the ΔNp63α–CYGB axis in HNSCC. We speculated that HNSCC cells employ a novel molecular mechanism to regulate resistance to bortezomib through the ΔNp63α–CYGB–ROS axis. This is the first time of using this approach to illustrate the mechanism of resistance to bortezomib in solid tumors, and to further explain the relationship between the upregulation of ΔNp63α in HNSCC and the increase in resistance to bortezomib. Regarding the regulation of CYGB, a previous study confirmed that non-coding sequence of CYGB promoter contains multiple conserved regions, and the genetic characteristics of these regions are often related to the response of hypoxic cells [39]. These genes include the hypoxia response element, the hypoxia-inducible protein binding site, and the hypoxia transcription factor recognition binding site. The transcription factors that have been proved to play a regulatory role include hypoxia inducible factor 1 (HIF1), stimulatory protein 1 (SP1), activator proteins (AP1, ap2), and nuclear factors (NF1, NFκB, NFAT) [40]. Our research showed that Δ Np63 α, as a transcription factor, is an important supplement to the regulation of CYGB.

In conclusion, we revealed a novel regulatory mechanism of ΔNp63α on bortezomib resistance: ΔNp63α promotes bortezomib resistance via the CYGB–ROS axis in HNSCC. The results may provide us a new strategy for the treatment of HNSCC with ΔNp63α as the target.

## Materials and methods

### Cell culture

UMSCC-11, UMSCC-17A, and UMSCC-17B were obtained from the University of Michigan. HN31 was obtained from Wayne State University. These cell lines used were pure (tested by STR profiling). All cells were authenticated and tested for mycoplasma contamination. These cells were grown in DMEM (Dulbecco’s modified Eagle’s medium) with 10% fetal bovine serum at 37 °C in a humidified atmosphere of 5% CO_2_ in air.

### Development of bortezomib-resistant cell lines

Cells were detached by trypsinization, counted, and seeded on 96-well plates at a rate of 10000 cells per well. The bortezomib-resistant HNSCC cell lines, 17B-R, were established from their parental line, UMSCC-17B, under continuous exposure to bortezomib (MCE, No.179324-69-7) in DMEM with 10% fetal bovine serum for over nine months. During this time, the concentration of bortezomib was increased stepwise weekly after confirmation of the maintained viability of the cells at the previous dose. After their establishment, the bortezomib-resistant cell lines were incubated in a bortezomib-free medium for 2 weeks, to confirm the stability of the resistance trait, and then subjected to all assays used in this study.

### Knockdown or overexpression of ΔNp63α

Knockdown of ΔNp63α was performed using Lentiviral mediated short hairpin RNA (shRNA) in HN-31 cells.

siRNA1 sequence: 5′-AGACTCAATTTAGTGAGCCACAGTA

siRNA2 sequence: 5′-TCCATGCCATCCACCTCCCACTGCA

siRNA2 sequence: 5′-CCACCTCCGTATCCCACAGATTGCA

Overexpression of Np63α was performed using pLenti-CMV-DeltaNp63-alpha-3FLAG-PGK-Puro in UMSCC-17B cells.

CMV-F: 5′-CGCAAATGGGCGGTAGGCGTG

MSCV-rev: 5′-CAGCGGGGCTGCTAAAGCGCATGC

### Tumor xenograft mouse model

Six-weeks old male BALB/c nude mice were purchased from Experimental Animal Center of Second Military Medical University (Shanghai) and maintained under specific pathogen-free conditions. Investigator was blinded to the group allocation during the experiment. Aliquots of 5 ×10^6^ cells were injected subcutaneously into each mouse for limiting dilution tumor formation. Tumor size was measured with calipers, and tumor volume was calculated according to the following formula: larger diameter × (smaller diameter)^2^/2.

### Western blotting and immunofluorescence

After the total cell extracts had been harvested and lysed on ice in RIPA buffer, they were loaded and resolved on sodium dodecyl sulfate–polyacrylamide gels and blotted onto PVDF membranes. Membranes were incubated with a primary antibody at 4 °C overnight. The dilution rates of the primary antibodies were 1:1000 for p63-α (Rabbit mAb, D2K8X-XP, CST, #13109), CYGB (Mouse mAb, Proteintech Group, 60228), β-actin (Servicebio, GB12001), GAPDH (Servicebio, GB12002). The secondary antibody was detected by Western ECL-enhanced luminol reagent (PerkinElmer Inc.).

For immunofluorescence, cells were seeded on sterile cover glasses placed in 6-well plates. After designated treatments, cells were fixed with 4% formaldehyde for 30 min, followed by permeabilization with 0.2% Triton X-100 for 5 min. Cells were washed with PBS and blocked with 5% BSA for 30 min, then incubated with primary antibodies at 4 °C overnight. The cells were washed again three times with PBS and incubated with secondary antibody. Subsequently, they were counterstained with 6-diamino-2-phenylindole (DAPI, Roche). An anti-fluorescence quenching reagent was used to stick the cell slide on the glass slide, and the setup was observed under a fluorescence microscope.

### RNA sequencing

RNA-seq libraries were prepared beginning with total RNA (RIN 8.60–10) extracted from control and ΔNp63α knockdownHN-31 cells, using RNeasy Plus Mini Kit (Qiagen, Valencia, CA). Quality of the reads was assessed using FASTQC. Relatively accurate and effective data are obtained by mass shearing through Trimmomatic. After RNA-seq sequencing evaluation, BCF tools was used to find out the possible SNP sites according to the mapping results, and SnpEff was used to determine the impact of SNP sites on genes. Deseq2 was used for gene expression difference analysis, and the expression difference analysis results were visualized. Cluster profiler was used for KEGG pathway and KOG classification enrichment analysis.

### Bisulfite sequencing PCR (BSP)

After DNA is treated using EZ DNA Methylation-Gold (ZYMO Research, D5005), BSP primers are designed to amplify the target fragment. And uracil (U) is completely transformed into thymine (T). The PCR product is sequenced to judge whether CpG site is methylated. An average methylation index (MtI) was calculated from the mean of CpG sites evaluated.

### Analysis of ROS levels

The HNSCC cells were treated with bortezomib and then collected to measure the ROS levels. To detect the ROS levels, we used DCFH-DA (Nanjing Jiancheng Bioengineering Institute, E004-1-1) by Flow Cytometry Set (ImageStream, Amnis). Flow cytometry analysis was done on an IDEAS software platform.

### Knockdown of CYGB by siRNA

Three sequences of CYGB small interfering RNA were designed as following: CYGB-homo-298, F: 5′-CCAUCCUGGUGAGGUUCUUTT-3′ R: 5′-AAGAACCUCACCAGGAUGGTT-3′ CYGB-homo-433, F: 5′-UCAACACUGUCGUGGAGAATT-3′ R: 5′-UUCUCCACGACAGUGUUGATT-3′ CYGB-homo-524, F: 5′-GGAACCGGUGUACUUCAAGTT R: 5′-CUUGAAGUACACCGGUUCCTT-3′. Lipo-3000 (Invitrogen, L3000008) was used to transfect siRNA in HN-31cells.

### CHIP assay

A total of 1 × 10^7^ proliferating HNSCC cells were used for the immunoprecipitation reactions. Antibody p63-α (Rabbit mAb, D2K8X-XP, CST, #13109) was used in the process. To perform the CHIP assays, the Magnify Chromatin Immunoprecipitation system (Invitrogen) was used according to the manufacturer’s instructions. The primers flanking the putative p63-binding sites used in the PCR had the following sequences:

Region1-Primer-F 5′-AACTTGAGCGCACCTCCGA-3′

Region1-Primer-R 5′-TTGTCTGCCAGGTCCTGTCT-3′

Region2-Primer-F 5′-GGAGGCAAATCCTTCCTCCT-3′

Region2-Primer-R 5′-CATCAAGTCTTGAGCGAGGA-3′

Negative-Primer-F 5′-CACTGGCAGTTGTCCAGAGA-3′

Negative-Primer-R 5′-TTCTGAGCCCAGAGTCCTGT-3′

Antibody: Anti-p63-α (D2K8X) XP (CST, #13109)

### Dual luciferase reporter assay

The CYGB promoter region (−1726 to –11) was amplified from human genomic DNA by PCR and subcloned into the pGL4.10 H352 vector linearized by XhoI/HindШ restriction (Promega). Luciferase activities of cellular extracts were measured 24 h after transfection using a Dual Luciferase Reporter Assay System (Promega, Madison, WI, USA). Data are presented as ratios between firefly and renilla activity. The primers used for cloning were as follows: pGL4.10(wt) F 5′-CTAGCAAAATAGGCTGTCCC-3′, R 5′CGTCTTCGAGTGGGTAGAATG-3′. pRL-CMV-Renilla luciferase vector.

### Statistical analysis

IBM SPSS 23.0 Statistics was used for statistical analysis. The data were presented as mean ± S.D. *P* values were calculated from Two-sided Student’s *t* test test or one-way ANOVA. GraphPad Prism 7.0 was used to calculate IC50 and graph. Repeated measures analysis of variance was used to compare the weight and tumor size of nude mice after tumor formation in different treatment groups. The Kaplan–Meier method was used to determine survival probability, and differences were assessed by the log-rank test. Spearman’s rank correlation test was used to analyse bivariate correlations. CYGB and ΔNp63α expressions in tumors isolated from nude mice tested by immunohistochemistry were calculated by the semi-quantitative H-score. All statistical tests were two-sided, and *P* < 0.05 was displayed as statistical significance.

## Supplementary information

Fig.WB-1

Fig.WB-2

Fig.S4

Fig.S6

Table 1

Reproducibility checklist

## Data Availability

All data generated or analyzed during this study are included in the main text and the supplementary information files.
