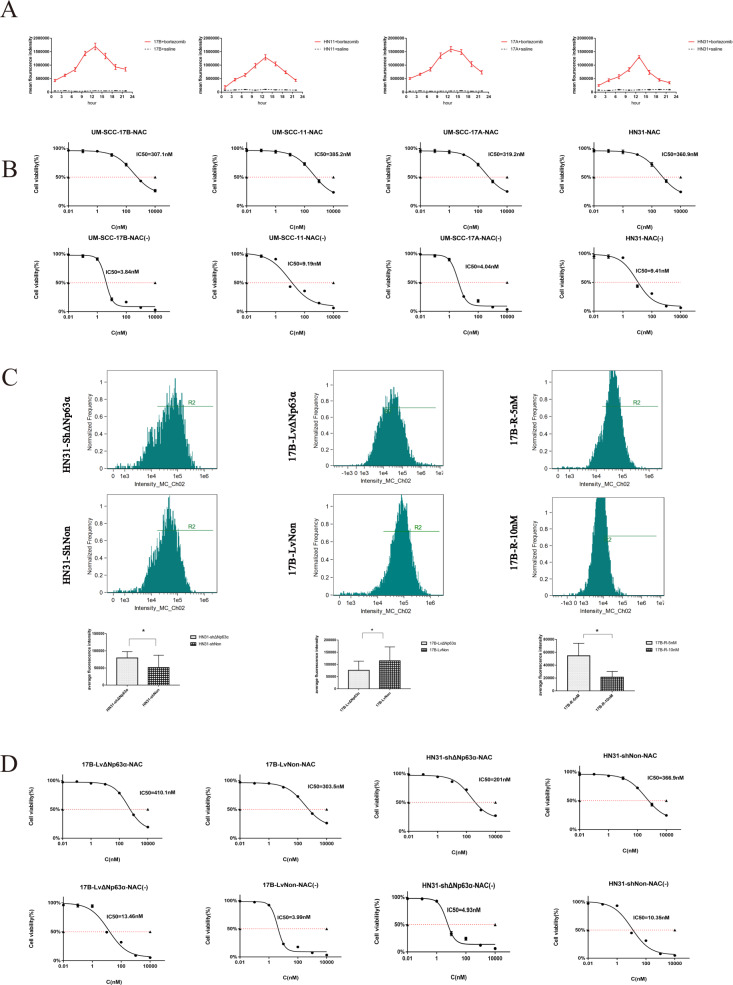# Correction to: ΔNp63α promotes Bortezomib resistance via the CYGB–ROS axis in head and neck squamous cell carcinoma

**DOI:** 10.1038/s41419-022-04870-1

**Published:** 2022-06-02

**Authors:** Peng Zhou, Caiyun Zhang, Xianmin Song, Dadong Zhang, Minhui Zhu, Hongliang Zheng

**Affiliations:** 1 Department of Otorhinolaryngology-Head and Neck Surgery, Changhai Hospital, Second Military Medical University, Shanghai, 200433 China; 2 Department of Otorhinolaryngology-Head and Neck Surgery, Affiliated Hospital of Xuzhou Medical University, Xuzhou, 221003 Jiangsu China; 3 3D Medicines Inc., Shanghai, 201114 China

**Keywords:** Predictive markers, Head and neck cancer

Correction to: *Cell Death and Disease* 10.1038/s41419-022-04790-0, published online 09 April 2022

The original version of this article unfortunately contained an error in Fig. 5. The authors found that the Fig. 5B and D (the second panel) in the online version are different from those in the version they submitted. We apologize for the error. The correct figure can be found below. The original article has been corrected.