# Supplementary figures and images for: ΔNp63α promotes Bortezomib resistance via the CYGB–ROS axis in head and neck squamous cell carcinoma
Source: Cell Death Dis. 2022 Apr 9;13(4):327. doi: 10.1038/s41419-022-04790-0 (PMC8994767; doi:10.1038/s41419-022-04790-0)

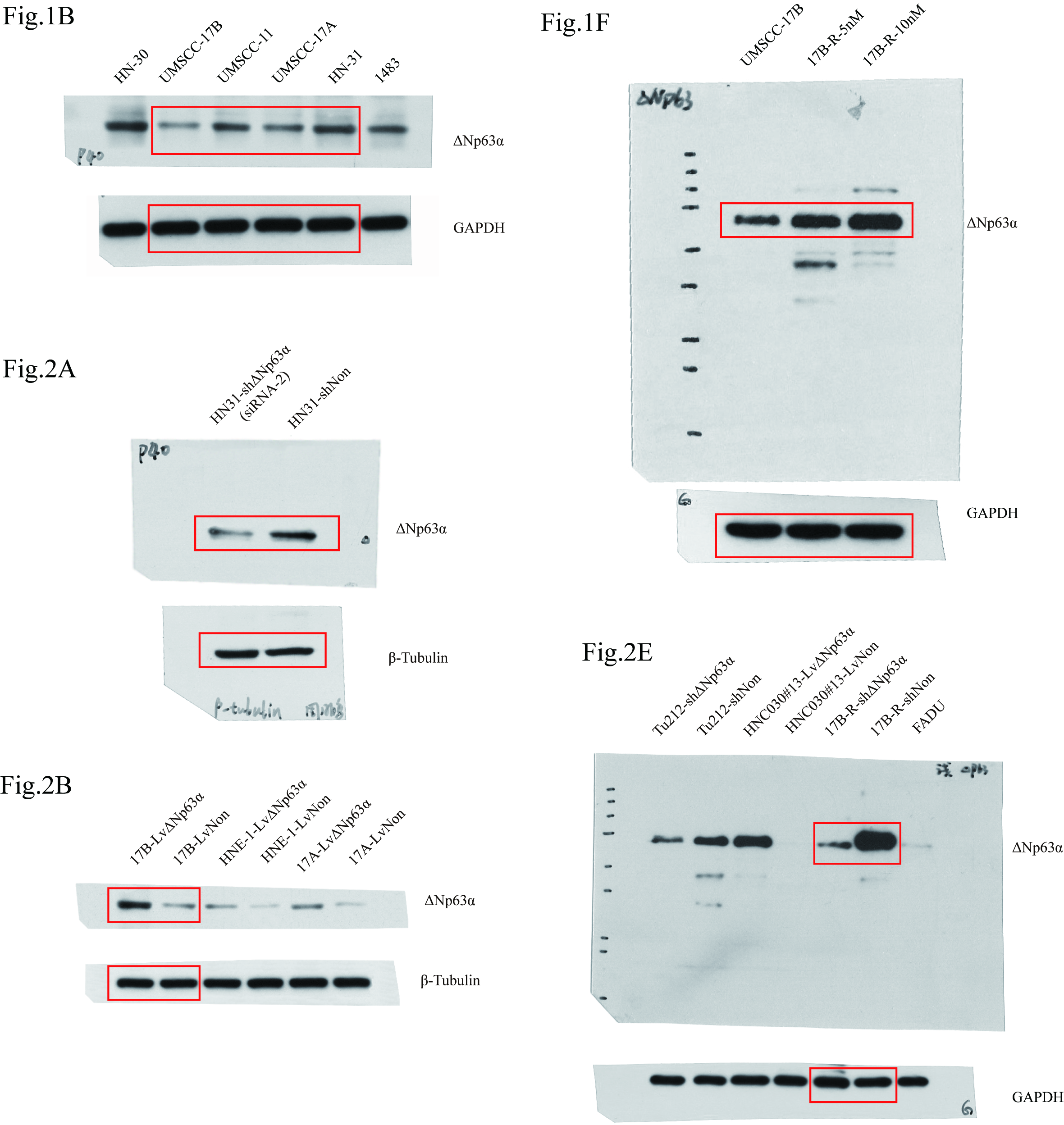

Supplement: Supplementary file 1 — Fig.WB-1 [file 41419_2022_4790_MOESM1_ESM.tif]

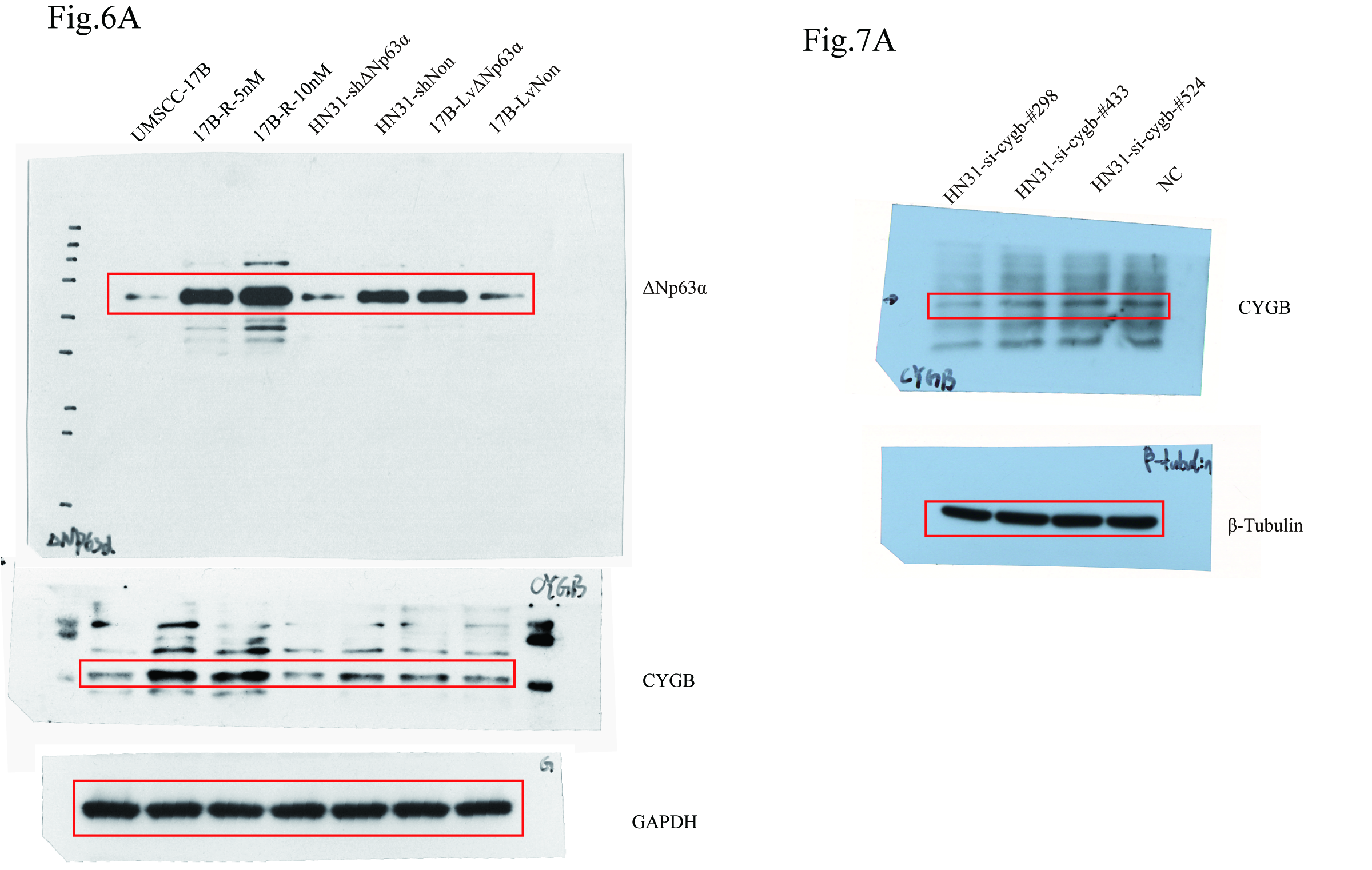

Supplement: Supplementary file 2 — Fig.WB-2 [file 41419_2022_4790_MOESM2_ESM.tif]

A

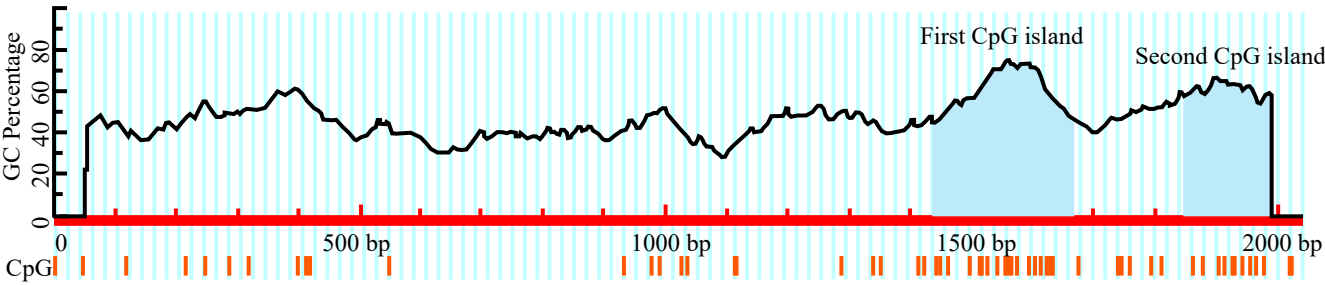

B

1st CpG island, HN-31

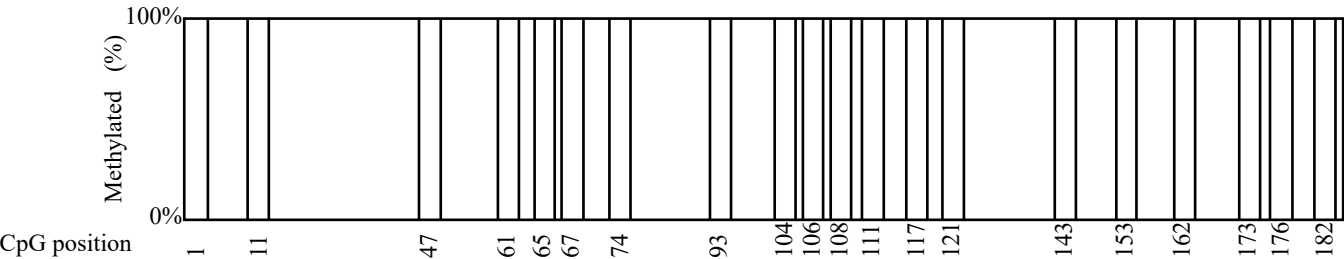

2nd CpG island, HN-31

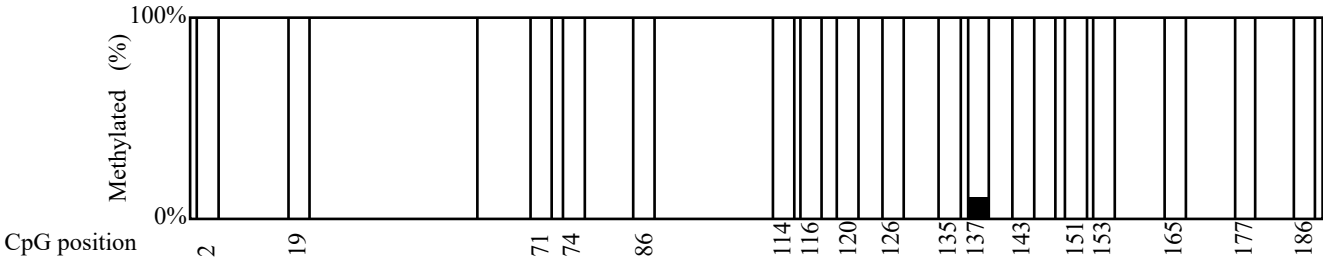

C

1st CpG island, UMSCC-17B

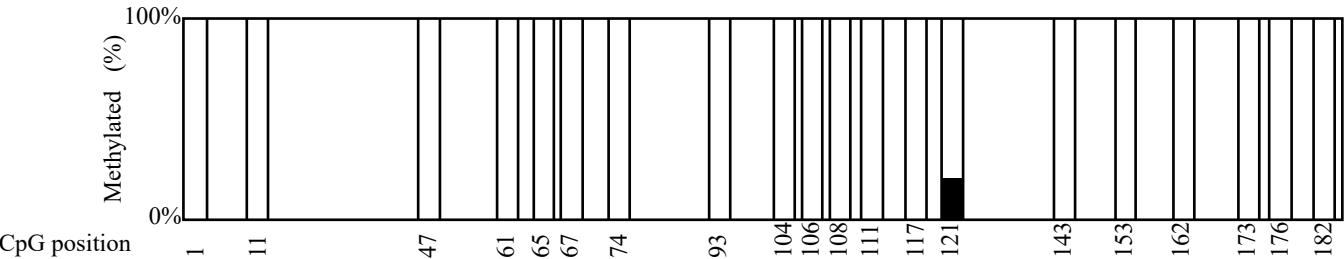

2nd CpG island, UMSCC-17B

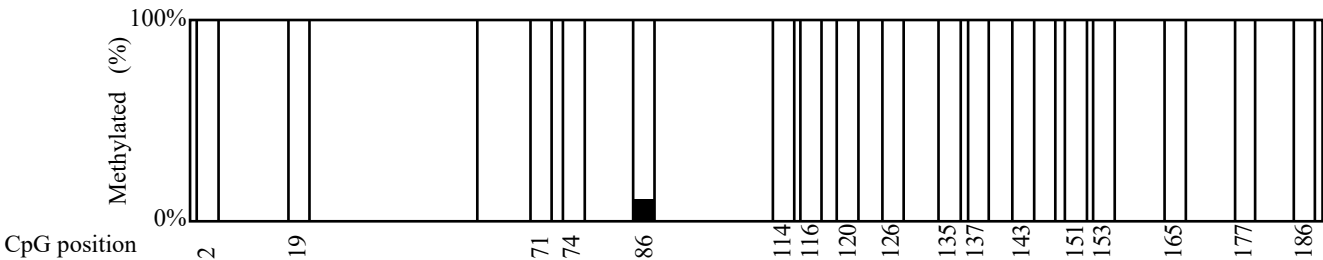

Supplement: Supplementary file 3 — Fig.S4 [file 41419_2022_4790_MOESM3_ESM.pdf]

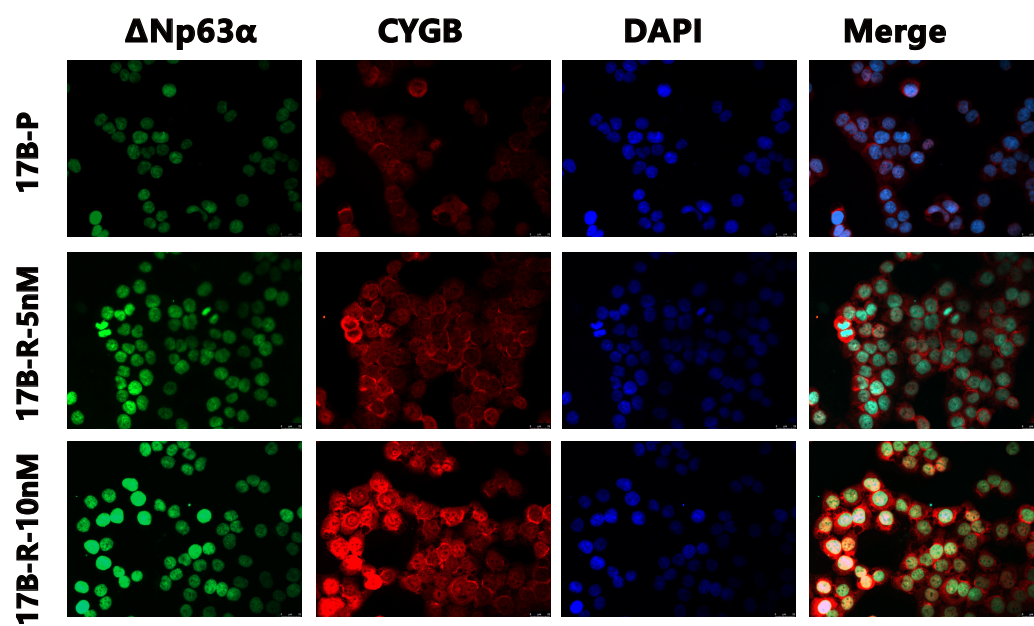

Supplement: Supplementary file 4 — Fig.S6 [file 41419_2022_4790_MOESM4_ESM.pdf]
