# Supplementary material for: ΔNp63α promotes Bortezomib resistance via the CYGB–ROS axis in head and neck squamous cell carcinoma
Source: Cell Death Dis. 2022 Apr 9;13(4):327. doi: 10.1038/s41419-022-04790-0 (PMC8994767; doi:10.1038/s41419-022-04790-0)
Supplement: Supplementary file 5 — Table 1 [file 41419_2022_4790_MOESM5_ESM.pdf]

Table1. Correlation analysis between ΔNp63α and CYGB expression in tumors of nude mice

	No.	ΔNp63α			*P*	*r*
	n	+	++	+++		
CYGB						
-	12	6	3	3	<0.001	0.464
+	30	8	10	12		
++	25	1	8	16		
+++	13	0	2	11		
